# Innovate or game over? Examining effects of product innovativeness on video game success

**DOI:** 10.1007/s12525-022-00521-7

**Published:** 2022-03-01

**Authors:** Franziska Handrich, Sven Heidenreich, Tobias Kraemer

**Affiliations:** 1 Faculty of Human and Business Sciences, Saarland University, Building C3 1, 66123 Saarbruecken, Saarland Germany; 2 Institute for Management, University of Koblenz–Landau, Universitätsstraße 1, 56070 Koblenz, Germany

**Keywords:** video games, product innovativeness, game innovativeness, innovation success, degree of newness, L86, O30

## Abstract

In recent years, the video game industry has invested significant amounts in the development of innovative elements for its products. However, it is still subject to debate whether these R&D investments always pay off. While prior research has confirmed that enhancing product innovativeness increases business performance in certain industries, corresponding findings for the video game market are lacking. This might be a result of missing theoretical conceptualizations and adequate empirical operationalizations of game innovativeness. Addressing this research gap, this study provides the first conceptualization and operationalization of game innovativeness, shedding first light on its performance effects. Based on longitudinal data on 351 computer games, our findings confirm that innovations in the game’s presentation and principle enhance short-term success, whereas innovations in a game’s storyline can be more of a hindrance than a godsend for companies. However, our results also show that performance effects of game innovativeness diminish over time.

**Supplementary Information:**

The online version contains supplementary material available at 10.1007/s12525-022-00521-7.

## Introduction

Innovative products or services form the basis of any successful company (OECD Oslo Manual 2005) and can possibly lead, in the long run, to the creation of new industries or markets (Malerba, 2007). Accordingly, several studies have confirmed that innovative firms have a higher performance in terms of total employment, employer attractiveness, market share, labour productivity as well as firm profitability (Cozzarin, 2004; Huang & Hou, 2019; Sommer et al., 2017; Ugur et al., 2018). Likewise, previous research has shown that implementing innovation-related business processes helps increasing corporate performance by creating competitive edges, thus improving market position and driving financial performance in the long run (Cozzarin, 2017; Hansen, 2014; Ngo & O'Cass, 2013). Within this respect, the degree of innovativeness as a proxy for innovations in a certain area represents an important factor for the success of new products and technologies, thus driving business performance (Gibb & Haar, 2010; Handrich, Handrich and Heidenreich 2015; Hügel et al., 2019). Prior research in this area has shown that product innovativeness can lead to the formation of new markets (Malerba, 2007) and is supposed to impact company performance positively (Gunday et al., 2011; Hubert et al., 2017; Ngo & O'Cass, 2013; Storz, 2008).

However, prior empirical research that focused on the linkage of product innovativeness and business success was restricted to companies in the service, manufacturing, transport, communication, construction or high-tech industry (Bhattacharya & Bloch, 2004; Chamberlin, Doutriaux and Hector 2010; Hagedoorn & Cloodt, 2003; Ngo & O'Cass, 2013; Patel & Pavitt, 1992). However, innovations can also be found in the entertainment industry and appear on corresponding markets in regular intervals (Marchand, 2016; Storz, 2008; Tschang, 2007). Especially the video game market as a cyclical platform market, is characterized by dynamic and volatile technical developments leading to disruptive innovations every several years (Koch & Bierbamer, 2016; Marchand, 2016). Nevertheless, achieving disruptive video game innovations requires a high degree of product innovativeness which always goes hand in hand with high investment costs. Therefore, managers and researchers alike ask themselves the question if these investments into R&D always pay off. While in other areas, such as manufacturing, transport, communication or construction, research demonstrated that product innovativeness is strongly related to success (Bhattacharya & Bloch, 2004; Chamberlin et al., 2010; Patel & Pavitt, 1992), empirical evidence on whether the product innovativeness-performance link (Kleinschmidt & Cooper, 1991) also exists in the video game industry is still missing. Yet, both empirical insights into whether investments in game innovativeness generally pay off, as well as into which innovated elements exhibit the strongest performance effects, could help companies to allocate their resources much more effectively. Furthermore, from a theoretical perspective, the confirmation of the product innovativeness-performance link in the video game industry, would extend its validity beyond traditional high-tech consumer products. However, up to now, studies concerning innovations in the video game industry focus upon innovation processes or dynamics of innovative systems (Jónasdóttir, 2014; Storz, 2008; Tschang, 2007), rather than on the degree of product innovativeness itself.

At least two factors have hampered progress in this respect. First, it lacks theoretical conceptualizations of game innovativeness as specific form of product innovativeness that accounts for the peculiarities of the video game market. Video games are complex entertainment products that essentially differ from other products as they combine audio-visual elements with complex modes of user engagement (Tavinor, 2008). Consequently, established conceptualizations of product innovativeness that were derived for other high-tech contexts are thus not directly adaptable. Second, perhaps as a consequence of missing conceptualizations, attempts to measure game innovativeness are also lacking. Yet, a good metric is required to establish a common ground that enables empirical investigations into antecedents and effects of game innovativeness. As a result, we know relatively little about the relationship between game innovativeness and success as no empirical study exists that investigates this linkage. Accordingly, the debate continues regarding whether high investments into R&D to enhance game innovativeness always pay off, leading up to our primary research question: How can game innovativeness be conceptualized and does it affect video game success?

To address these issues, this study first derives a detailed theoretical conceptualization of game innovativeness. According to prior literature, a game’s presentation, principle and storyline represent the main components of any game and as such the most important starting points for innovations in video games that might drive product success (Engelstätter & Ward, 2013; Storz, 2008; Tschang, 2007; Wood et al., 2004). Consequently, we conceptualize game innovativeness as higher-order concept, that encompasses three constituting elements: (1) game principle innovativeness refers to the degree of innovativeness present in the interaction between the game and the player, whereas (2) storyline innovativeness relates to the degree of innovativeness in a game’s story, challenges or campaigns, and (3) presentational innovativeness reflects the degree of innovation in visual and auditory features of the game. Based on this conceptualization, theoretical rationales on whether and how game innovativeness affects video game success are derived. Afterwards, we conduct a large scale, longitudinal analysis to assess whether and to what extent game innovativeness also represents an important performance driver for the video game industry. More specifically, sales data of 351 computer games published between 2012 and 2015 were collected to measure video game success. As Moore’s law (Moore, 1965) suggest that 18 months is the average point in time when new technical capabilities appear on the market, we differentiated short-term success, encompassing the number of all sold items in Europe during the first 18 months, from long-term success, encompassing the number of all sold items in Europe during the 19th-36th month. This secondary data for the dependent variables was then combined with primary data on the independent variables to avoid common method bias. Specifically, expert judges evaluated the 351 games concerning their degree of innovativeness of presentation, game principle and storyline using established measurement items. Finally, partial least squares (PLS) structural equation modeling (SEM) was used to examine the effects of the degree of innovativeness in video games’ presentation, game principle and storytelling on the short-term and long-term success.

The manuscript is structured as follows. First, the conceptual background of our study is laid out before explicit hypotheses are derived. In the following sections the empirical part of the manuscript begins with information on the date and applied procedures as well as the discussion of applied statistical methods and corresponding results. In the concluding sections, we discuss implications of the achieved findings, potential limitations and possible avenues for future research.

## Conceptual background

### Product innovativeness and new product success

Nowadays, more and more markets can be considered as being highly competitive, forcing companies to continuously launch new products or services to counteract the increasing pressure of their competitors (Fang, 2008; García-Cruz et al., 2018), to keep their current market positions (Kuester et al., 2012) and survive in the long run (Handrich et al., 2015). One common approach concerning the successful introduction of new products and thus to achieve sustainable competitive advantages is enhancing the degree of product innovativeness. Product innovativeness can further be specified as the degree of how much the developed products differ from other competitive products already on the market and to which degree these newly developed products include radical new ideas (Fang, 2008; Hilmi et al., 2010). As such, product innovativeness is closely linked to creativity (Su et al., 2013). More specifically, “it depends on the amount and creativeness of new knowledge used to develop new products” (Su et al., 2013, p. 474). The more creative the knowledge used in the new product development process is, the higher the resulting product innovativeness can be (Moorman & Miner, 1997; Su et al., 2013). While creativity and product innovativeness are thus somewhat interrelated, creativity alone represents only a necessary but not sufficient condition to reach product innovativeness within the new product development process as other factors also play an important role (Valgeirsdottir et al., 2015). Accordingly, within this study creativity is seen as input in the new product development process that strongly determines “the extent to which the product is different from competing alternatives in a way that is valued by customers” (Sethi et al., 2001, p. 74), that is product innovativeness as output.

While most research concerning product innovativeness is focused upon the effect on performance measures (Hult et al., 2004), other studies concentrate on the differences between innovativeness of services and goods and their effects on customer satisfaction (Stock, 2011) or consumer resistance (Heidenreich & Kraemer, 2016). All in all, product innovativeness as a prerequisite of producing radical innovations is widely seen as key success factor of companies (Hult et al., 2004). More specifically, product innovativeness is known to be an antecedent of gaining competitive advantages, ensuring long-term growth and thus increasing overall business performance (Hult et al., 2004; Stock, 2011). Therefore, it is important for companies to understand to which degree it is best to increase product innovativeness in order to enhance the probability of a successful market introduction and new product’s success (Kuester et al., 2012).

Since, the video game industry can be described as a very dynamic and competitive market with short product lifecycles and continuous introductions of new technologies (Cenamor et al., 2013; Jónasdóttir, 2014; Subramanian et al., 2011; Williams, 2002), innovations are also necessary to achieve market results and compete with other companies for market share (Jónasdóttir, 2014; Situmeang et al., 2016). Subsequently, product innovativeness as a precondition of successful innovation development should also play an important role in the video game industry to gain competitive advantages and to increase game success. However, current literature is lacking empirical proof that product innovativeness, or in this specific case game innovativeness, is also a key driver for game success. A possible explanation, why empirical validations are missing, might lie in the specific characteristics of video games. As a unique entertainment product, video games essentially differ from other products as they combine audio-visual elements with complex modes of user engagement (Tavinor, 2008). Consequently, established conceptualizations and measures of product innovativeness that were derived for consumer high-tech products and the like do not account for the specific peculiarities of video games and thus are not directly adaptable. As a consequence, we will first conceptualize game innovativeness based on existing literature from the video game industry in the following chapter before executing an empirical evaluation of the developed concept afterwards.

### Conceptualizing game innovativeness

For the conceptualization of game innovativeness, the most important areas for innovations in video games have to be identified. Studies of Engelstätter and Ward (2013), Storz (2008), Tschang (2007) and Wood et al. (2004) always emphasize that presentation, game principle and storyline are the main components of any game and therefore have also been defined as important starting points for innovations in video games. In the following, we will shortly define these gaming components before evaluating their potential for innovation and thus suitability as constituting elements of game innovativeness.

The presentational aspects of a game include visual and auditory features, which are responsible for the look and sound the player experiences during the game (King et al., 2010). Previous research identified presentation as the main aspect that distinguishes one game from another (Tschang, 2007). Moreover, researchers found that good graphics and sound make games appear more realistic (Hofacker et al., 2016) thus increasing the degree of player immersion (Boyle et al., 2012; Hofacker et al., 2016). In addition to that, research concerning the preferences of players pointed out, that players rate realistic graphics and sound effects as the most important features of video games (King et al., 2010; Nacke et al., 2010; Wood et al., 2004) due to the fact that good presentation leads to a better gameplay experience (Nacke et al., 2010). Especially professional gamers focus on technical increases of sound and graphics, next to new possibilities of technical speed and media access which new platforms may offer (Subramanian et al., 2011). Accordingly, in recent years, the industry has seen an increase in technical possibilities concerning sound and graphical aspects (Gallagher & Park, 2002; Schilling, 2003). Auditory features went from bare bleeps to simple melodies resulting in 3D soundtracks recorded by actual orchestras (Paterson et al., 2010). With regard to innovation of graphical features of games, there have been around five different stages of technical innovations within the video game industry (Gallagher & Park, 2002). As the increased processing power of platforms allowed for more and more complex graphical features to be embedded in games (Situmeang et al., 2016), the level of player immersion, involvement and arousal increased as well (Boyle et al., 2012). In line with these arguments, the authors conclude that presentation is not only an important aspects of video games, but should also be closely regarded as constituting element of game innovativeness.

The second important element of video games is the game principle. Game principle refers to the interaction between the game and the player, how the player can advance within the game (Tschang, 2005) and the basic rules applied in the game (Hofacker et al., 2016; Salen et al., 2004), for example if the game involves solving puzzles, combining certain elements or fulfilling specific quests (Wood et al., 2004). Moreover, the right method of game feedback and player control (Tschang, 2005), as well as the right level of challenge (Desurvire & Wiberg, 2008; Hsu & Lu, 2007), are important drivers of good user experience (Desurvire & Wiberg, 2009; Hofacker et al., 2016) and therefore also belong to the element of game principle. Consequently, innovations in the game principle are often brought forward by successful video games (‘Superhot’, ‘Wii Sports’), such that it will be also considered as constituting element of game innovativeness.

The third innovational aspect relates to storylines of video games. The storyline provides the background setting for a game including elements like story, challenges or campaigns (Lin et al., 2012; Wood et al., 2004). Likewise, it attracts the player and keeps him or her attached throughout the game (Lin et al., 2012). Often games are based on stories that have a link to other media, for example movies or books, thus offering the players the chance to expand their experience and engage personally in the virtual world (Aoyama & Izushi, 2003). Some researchers have already identified the importance of a storyline for video games. Schneider (2004) found, that the level of immersion increases for players of first-person shooters as soon as a storyline was included within the game. Similarly, Wood et al. (2004) pointed out that real-life settings of video games enhance the feeling of immersion for players and thus are important for games’ success. Moreover, the narratives of video games are supposed to be key factors for successful video games (Harper, 2011) as they provide relevance and meaning to the game play experience (Hofacker et al., 2016). Consequently, as shown above, innovations in the area of storyline are most common in the video game industry such that storyline will also be included as constituting element of game innovativeness.

All in all, theoretical rationales outlined above lead to the assumption that presentation, game principle and storyline are the constituting elements of game innovativeness. However, empirical evidence on whether and how game innovativeness influences video game success, and which of the previously identified elements might be most important in this regard, is still lacking. As a consequence, we will deduce the theoretical rationales for potential effects of the degree of innovativeness for presentation, game principle and storyline on game success in the following chapter, before conducting the empirical validation.

## Hypotheses development

As studies in other areas have demonstrated, the effect of product innovativeness on firm performance can change over time (Zhao & Roy Dholakia, 2009). Based on the findings of the Kano model (Kano 1984; Dubey et al., 2019), customers classify specific characteristics of products in basic needs, so-called “must-haves”, and in attributes, which are normally not expected, but delight the customer (Ludwig et al., 2017), so-called “delighters” (Kim & Yoo, 2020). Over time, the so-called “delighters” which might have been a buying factor for the customer, become standards in the industry and are then classified as basic requirements (Matzler et al., 1996; Lin et al., 2017). For example, in the beginning of mobile phones, the battery life was short and having a mobile phone with several hours of power an exception. However, nowadays in the era of smartphones a good battery life is a basic requirement and met with customer dissatisfaction if not available. Moreover, such changes in the perception of product attributes are not limited to specific industry segments, but can occur in all customer-buying situations (Matzler et al., 1996). For instance, contrary to the example of the mobile phones, the effect of the degree of innovativeness on product performance might also start with being insignificant and switch to having a positive impact in the long-term perspective. A good example here is the integrated internet connection of television sets. In the beginning, only a handful of services for television sets were available, thus the functionality did not provide an added service to the customer and subsequently did not influence short-term success. However, the more online services especially designed for television sets, for example video-on-demand services, came online, the more important the innovation of integrated internet connection for product success became. Consequently, in line with other industries, a longitudinal investigation on whether and how game innovativeness influences video game success over time. Therefore, the subsequent theoretical derivation of hypotheses differentiates whether each of the constituting elements of game innovativeness have an impact on short-term vs. long-term success.

Game presentation contains the visual and auditory aspects, which account for the look and sound the customer experiences while playing the game (King et al., 2010). Overall, innovations of increasing presentational features are providing both developers and players with opportunities to create new challenges and thus new industry segments in the long run (Gallagher & Park, 2002). Moreover, the creation of new graphical and auditorial possibilities can be classified during the market entry as a delighter functionality for the game, whereas over time, this functionality will become a new standard, or in terms of the Kano model a “must have” for succeeding games (Dubey et al., 2019). An example for such a new feature which became an industry standard is 3D graphics, which first appeared on the market around 1982 with the game “Battlezone” (Atari), and nowadays is seen by game players as a standard function for any successful video game (Storz, 2008; Wood et al., 2004). Therefore, over time as delighters translate to basic functions, customers get used to features as standard functionalities and subsequently the former innovative feature does not have an impact on the buying decision anymore (Anderson et al., 1994). This reasoning leads the authors to the following hypothesis:

- H1: *Presentation innovativeness exerts a positive impact on short-term game success (H1a), while it does not have an impact on long-term game success (H1b).*

The second important game characteristic is the game principle, which explains the interaction between the game and the player, how the player can advance within the game (Tschang, 2005) and the basic rules of the game (Hofacker et al., 2016; Salen et al., 2004). Both developers and users alike focus upon games with good game principles (Tschang, 2005) as an innovative game principle forms the basis for increasingly immersive games (Tschang, 2007). Moreover, Cox (2014) suggests, that optimizing the game principle leads to an optimization of player experiences and consequently to an increase of sales. However, concerning the development of the effect of game principle on game success over time, we expect that there will be no difference between the effects on short-term and long-term game success. In line with other guiding principles, like the structured story approach for books or motion pictures which clearly mostly follows a certain pattern (Clark, 1996; Freytag, 1894; Gee & Kegl, 1983), game principle is a timeless and classic element of video games. Moreover, recent releases of so-called retro games like the game console Nintendo SNES mini and the huge rush on it (Nintendo, 2019), clearly show, that games with a good game principle survive on the market for several decades. Hence, the following hypotheses can be suggested:

- H2: *Game principle innovativeness exerts a positive impact on short-term game success (H2a), while it also has a positive impact on long-term game success (H2b).*

Finally, the third important aspect of games, storyline, provides games with the background setting, including elements like story, challenges or campaigns (Lin et al., 2012; Wood et al., 2004). Previous studies in the video game industry have demonstrated that an improvement of the storyline of a game can lead to an increase of player immersion. Consequently, this is one of the main motivators for customers to play games (Williams et al., 2008) and hence buy the game. Moreover, in line with the Kano model, a good storyline innovativeness will first be identified by customers as a delighter aspect of the game, thus leading to great delight and satisfaction for the customer. Then, over time, the storyline will translate into a standard must-be requirement, which is a prerequisite for the customer during the buying process, but does not offer additional satisfaction if fulfilled (Matzler & Hinterhuber, 1998; Dubey et al., 2019; Zhao & Roy Dholakia, 2009). Consequently, we propose that:

- H3: *Storyline innovativeness has a positive effect on short-term game success (H3a), while it has no effect on long-term game success (H3b).*

## Data and procedures

The data set was collected in 2017 and contains primary and secondary data on 351 computer games published between 2012 and 2015 (for the distribution of release dates please see Online Appendix A). In line with other research done in the video game industry (Cox, 2014; Situmeang et al., 2016; Wesley & Barczak, 2016), we decided to measure game success through sales figures as they are not affected by different market prices and differing exchange rates within the European countries. Consequently, we collected for each of the 351 games weekly sales figures from the *vgchartz-database* for the first three years after market introduction. Due to the fact that based on Moore’s law every 12 to 24 months a jump in technical performance is happening we decided to use 18 months as the average point in time when new technical capabilities appear on the market and might initiate a new generation of games (Moore, 1965). Consequently, the dependent variable of video game success was differentiated into short-term and long-term success by measuring the number of all sold items during the first 18 months (short-term success) and second 18 months (long-term success) after market introduction. As shown in Online Appendix A, the release date varies throughout the year from January to December. Hence, no systematic assignment of 1 or 2 Christmas seasons (where video sales numbers of video games might be higher compared to the rest of the year) can take place based on the differentiation in first 18 months (short-term success) and second 18 months (long-term success) after market introduction, which would be the necessary condition for a systematic bias in our dependent variable. To operationalize the degree of game innovativeness, we utilized some items from the product innovativeness scale by Gatignon and Xuereb (1997) and adapted them to the context of the video game industry (for exact wording of the items please see Table 1). All games were evaluated by eight independent expert judges, which is common practice in marketing and innovation research (Sweeney & Soutar, 2001; Zaichkowsky, 1985) - concerning their degree of innovativeness of presentation, game principle and storyline using the respective measurements. More specifically, we approached a renown e-sport clan in Germany and recruited eight expert raters (average age of 30.38 years) for our evaluations. The expert raters had on average 23 years of gaming experience (at least 20 years), played 12.88 hours a week on average and reported a very high involvement with video games (6.13 with 7 representing the maximum value). We instructed the expert reviewers to use the internet to gather information about each of the 351 games in order to exclude any distortion effects that might have come up as a result of priming the expert raters by giving them preselected information. More specifically, we asked the expert raters to use the gathered information to rate the games’ innovativeness dimensions relative to the market conditions present at the time of the release in order to exclude any distortion effects that might have come up due to the time elapsed since release of the game. As a result the expert rater assessment is based predominantly on secondary data, not on a personal experience. Each expert rater then evaluated each game by means of the selected items. The final value of the construct ‘game innovativeness’ was calculated by building the average of all expert opinions, making sure that no statistical outliers manipulated the results of this study.^1^

**Table 1:** Operationalization of game innovativeness

Items to measure the different aspects of game innovativeness		
Presentation Innovativeness	The presentation (graphics and sound) of the game …is very innovative - the first of a kind on the market. …differs radically from already existing games on the market. …can be described as revolutionary.	Items 3.-6. of the Innovation scales from Gatignon and Xuereb (1997) were adapted to fit the area of video games
Game Principle Innovativeness	The game principle of the game …is very innovative - the first of a kind on the market. …differs radically from already existing games on the market. …can be described as revolutionary.	
Storyline Innovativeness	The storyline of the game …is very innovative - the first of a kind on the market. …differs radically from already existing games on the market. …can be described as revolutionary.	

As other game characteristics have been identified to influence short-term and long-term game success of entertainment products by prior research (Marchand, 2016; Storz, 2008; Tschang, 2007), we implemented brand and developer popularity, production costs, platform availability and game series as control variables in this research model to test the hypotheses more thoroughly. Brand and developer popularity were measured with seven levels of popularity from unknown to well-known, also assessed by the expert panel. Production costs were retrieved from a German gaming magazine website (www.pcgameshardware.de) and measured in Euros. Available platforms was measured as the number of platforms available for purchase in case of each game. Being part of a game series finally was measured as a dichotomous variable with “yes=1” and “no=0”.

## Analysis and results

**Fig. 1:**
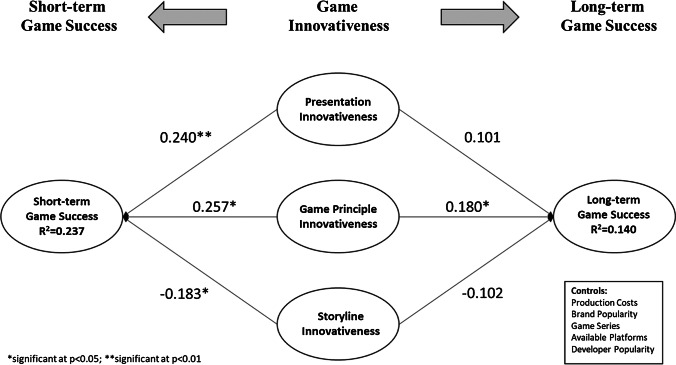
Structural Model Results

SmartPLS 2.0 was applied to empirically investigate the hypotheses based on the following considerations. Partial least squares (PLS) structural equation modelling (1) is often applied for models based on the prediction of possible relationships, (2) does not require normally distributed data and (3) the required sample size especially for complex research models in PLS is significant smaller compared to covariance-based methods (Chin & Newsted, 1999). To test the hypotheses, we evaluated the path coefficients and their significances with a centroid-weighting scheme and mean replacements for missing values. The resulting parameter estimates can also be seen in Fig. 1. Moreover, we employed nonparametric bootstrapping with 5000 replications as well as individual changes processing to estimate the standard errors (Chin, 1998).

The assessment of this PLS model and its hypotheses was conducted following the evaluation process of Anderson and Gerbing (1988). In a first step, we tested the measurement model with the focus on its reliability as well as validity. In the second step we evaluated the model for its main effects. As the measurement model consists of reflective constructs, we began the examination with an exploratory principle component analysis to test for content validity of the constructs. Each loading turned out to be above the recommended threshold value of 0.70 (Homburg & Giering, 1996). Next, we evaluated the model for indicator and construct reliability. Again, we can confirm that indicator and construct reliability exist, as all indicator loadings (see Table 2) as well as the composite reliability (see Table 2) indicate (Bagozzi & Yi, 1988; Chin, 2010; Nunally & Bernstein, 1994).

**Table 2:** Game innovativeness - Measurement model fit

Constructs	Item label	Mean	SD	Loading	Significance (t-value)	Significance (p-value)
Presentation Innovativeness	Present_1	3.817	0.722	0.990	932.223	0.000
	Present_2	3.701	0.694	0.991	1099.511	0.000
	Present_3	3.488	0.703	0.993	1458.369	0.000
Game Principle Innovativeness	Game_Prin_1	3.676	0.728	0.987	790.233	0.000
	Game_Prin_2	3.617	0.729	0.988	757.283	0.000
	Game_Prin_3	3.397	0.727	0.992	1229.680	0.000
Storyline Innovativeness	Story_1	3.641	0.925	0.994	840.803	0.000
	Story_2	3.579	0.893	0.995	1510.606	0.000
	Story_3	3.358	0.855	0.996	2293.057	0.000

Furthermore, we applied the Fornell and Larcker (1981) criterion to evaluate whether discriminant validity is given among the constructs of this model. We found no problems with respect to discriminant validity (see Table 3), as all the constructs exhibited AVE values above the critical threshold of 0.5. Furthermore, the corresponding square root was bigger than all the correlations with other constructs (Fornell & Larcker, 1981). Finally, we calculated the Q^2^-value for the dependent variable to test for predictive validity. The results yield Q^2^-values above the critical threshold value of 0 thus predictive validity can be confirmed (Geisser, 1974; Stone, 1974). In summary, these results confirm that the constructs provide a good measurement model fit and that we can continue with the evaluation of the structural model.

**Table 3:** Test for discriminant validity of constructs

	Presentation Inn.	Game Principle Inn.	Storyline Inn.
Presentation Inn.	0.979		
Game Principle Inn.	0.715	0.983	
Storyline Inn.	0.672	0.592	0.990

(squared correlations among constructs with AVE on the diagonal)

We assessed the hypotheses by evaluating the path coefficients and their significances. A summary of the results can be found in Fig. 1 and Table 4.

**Table 4:** Results on hypothesized relationships and controls

	Hypothesis	Hypothesized direction	Path coefficient	Significance t-value (p-value)	Hypothesis test
Main Effects	Presentation Innovativeness →Short-term Success (H1a)	Positive	0.240	2.645 (0.008)	Confirmed
	Presentation Innovativeness →Long-term Success (H1b)	No effect	0.101	1.023 (0.306)	Confirmed
	Game Principle Innovativeness →Short-term Success (H2a)	Positive	0.257	2.336 (0.020)	Confirmed
	Game Principle Innovativeness →Long-term Success (H2b)	Positive	0.180	2.460 (0.014)	Confirmed
	Storyline Innovativeness → Short-term Success (H3a)	Positive	-0.183	2.244 (0.025)	Rejected
	Storyline Innovativeness → Long-term Success (H3b)	No effect	-0.102	1.200 (0.230)	Confirmed
Controls	Production Costs → Short-term Success	-	0.011	0.127 (0.899)	-
	Production Costs → Long-term Success	-	-0.064	1.583 (0.112)	-
	Brand → Short-term Success	-	0.162	3.753 (0.000)	Positive effect
	Brand → Long-term Success	-	0.155	4.052 (0.000)	Positive effect
	Game Series → Short-term Success	-	0.164	5.081 (0.000)	Positive effect
	Game Series → Long-term Success	-	0.103	2.949 (0.003)	Positive effect
	Avl. Platforms → Short-term Success	-	-0.095	2.043 (0.041)	Negative effect
	Avl. Platforms → Long-term Success	-	-0.083	1.263 (0.207)	-
	Developer → Short-term Success	-	0.029	0.490 (0.624)	-
	Developer → Long-term Success	-	0.133	2.500 (0.012)	Positive effect

The results confirm a good model fit with an adjusted R^2^ for short-term success of 0.237 and long-term game success of 0.140. Furthermore, we calculated the Variance Inflation Factor (VIF) values for the complete structural model. As the highest VIF value was 2.59, we can conclude that no multicollinearity exists (Henseler et al., 2009). H1a predicted that presentation innovativeness has a positive impact on short-term game success. This hypothesis is supported as the results indicate a positive effect of presentation innovativeness on short-term success (H1a: β= 0.240, *p*<0.01). Moreover, we also found support for hypothesis H1b, as the presentation innovativeness has no effect at all on long-term game success (H1b: β= 0.101, n.s.). In support of H2a, we found a positive effect of game principle innovativeness on short-term success (β= 0.257, *p*<0.05). Likewise, H2b is supported as the expected positive relationship between game principle innovativeness on long-term success is significant (β= 0.180, *p*<0.05). Finally, we did not found support for hypothesis H3a as the expected positive relationship between storyline innovativeness and short-term success is negative (H3a: β= -0.183, *p*<0.05), whereas for long-term success the results show that storyline innovativeness has no effect on long-term game success at all (H3b: β= -0.102, n.s.). Consequently, we found some support for hypothesis H3b. Hence, all in all, we found support for the positive influence of presentation and game principle innovativeness on short-term success and for the latter also a significant effect on long-term game success. Concerning storyline innovativeness there seems to be a negative impact on short-term success, which in the long run is not relevant anymore. The control variables production costs (β=0.011, n.s.; β=-0.064, n.s.) and platform (β=-0.095, *p*<0.1; β=-0.083, n.s.) have neither an influence on the short-term nor long-term success of video games. At the same time, brand popularity (β=0.162, *p*<0.01; β=0.155, *p*<0.01) and game series (β=0.164, *p*<0.01; β=0.103, *p*<0.01) were positively related to short-term and long-term success. Finally, developer popularity only affected long-term success (β=0.029, n.s.; β=0.133, *p*<0.05)

## Discussion

### Summary of findings

Nowadays, in almost every market, companies are facing tremendous competition. To counteract the danger of being left behind in such fast-evolving markets, firms are utilizing the power of innovations to fight off their competitors (Lampel & Shamsie, 2000; O’Hern & Rindfleisch, 2010). As a result, the development and introduction of innovative products has been identified as an important factor for securing and contributing to companies’ success in the long run (Gunday et al., 2011; Hubert et al., 2017; Ngo & O'Cass, 2013; OECD Oslo Manual 2005; Storz, 2008). The findings of this empirical research produced several notable findings.

First, the empirical results concerning the impact of the degree of innovativeness of presentation on short-term success are in line with prior studies done in the video game industry concerning player preferences, which indicated that presentation was important for short-term success in general (Nacke et al., 2010; Wood et al., 2004). However, the results clearly depict that the degree of innovativeness of presentation does not play a role for long-term business success. As Moore’s law (Moore, 1965) predicted, there is currently an increase in technical performances of computers every 12 to 24 months. Subsequently, this results in the production of new games based on these new technical capabilities. Hence, presentation does not have a long-term innovational impact, as the lifecycle of games with only good graphics and sound is so small that it cannot have an impact on long-term company success.

Second, our results demonstrate that the degree of innovativeness in game principle is relevant for the short-term game success. This result is in line with previous research conducted on important factors of video games rated by players. Wood et al. (2004) describes in his study that good game principle, the so-called nature of games, was rated by game players as a popular characteristic of video games, which explains the positive impact of innovative game principle of short-term success. Similarly, earlier studies have reported that successful games also include a good balance between challenge and achievements, as challenging tasks and positive feedback are the key to increase the player experience (Przybylski et al., 2010). Thereby, players can fully concentrate on the general tasks of the games and immerse themselves fully into the game (Hsu & Lu, 2004). However, contrary to our expectations, the degree of innovativeness in game principle did not significantly affect long-term game success. In our opinion, this might be a result of the fact that after a certain time on the market the game principle of the once innovative game has been copied or adapted by other competitors, thus decreasing the competitive advantage of the game principle (Koellinger, 2008). As a result, the effect of innovative game principles on long-term game success is not significant.

Third, contrary to hypothesis 3a, the degree of innovativeness within the storyline negatively impacts short-term game success, which goes against common understanding that game players favor video games with innovative stories and background settings (Wood et al., 2004). However, these findings suggest that with respect to storyline, consumers might expect settings similar to those they are used to. Accordingly, consumers seem to prefer storylines similar to their established video game portfolio trying to preserve their established status quo (Zaltman & Wallendorf, 1983). This explains why many successful video games are part of established game series based on the same kind of storyline (Marchand & Hennig-Thurau, 2013), e.g. Grand Theft Auto (Rockstar Games). Contrary to the negative effect found in testing hypothesis 3a and in line with hypothesis 3b, storyline innovativeness was, however, not important for long-term success.

### Theoretical implications

The findings of this study may contribute to the current knowledge about innovativeness of video games in several ways. First, effects of product innovativeness in other industries, namely service, manufacturing, transport, communication, construction or high-tech sectors, are well researched and widely known (Bhattacharya & Bloch, 2004; Chamberlin et al., 2010; Hagedoorn & Cloodt, 2003; Ngo & O'Cass, 2013; Patel & Pavitt, 1992). However, corresponding research within the video game industry up to now has only focused upon topics like the innovation process, dynamics of innovative systems and creativity processes (e.g. Jónasdóttir, 2014; Storz, 2008; Storz, 2008; Tschang, 2007) but not on the degree of innovativeness of specific game characteristics and their impact on short-term and long-term game success.

Second, one key implication of this study is the applicability of the theory of the Kano model in the highly dynamic and competitive market of video games. The video game industry is based upon continuously changing technologies (Cenamor et al., 2013; Jónasdóttir, 2014; Subramanian et al., 2011; Williams, 2002) with which companies compete with other companies for customer satisfaction and ultimately for market share (Jónasdóttir, 2014; Situmeang et al., 2016). Our findings confirm, that game principle as well as presentation innovativeness function as delighters (Kim & Yoo, 2020) when it comes to video game sales. More specifically, both types of game innovativeness have strong positive effects on short-term success, however, this effect diminishes over time, being rather small but significant for game principle innovativeness on long-term success, and still positive but insignificant for presentation innovativeness on long-term success. Consequently, this study provides first evidence, that the Kano model is also valid for the video game industry.

Third, this research model and its results offer further support for the confirmation\disconfirmation paradigm, which states, that the customer is only satisfied when the product experience is in accordance to the customer’s expectations (Herrmann et al., 1999; Homburg & Stock-Homburg, 2006). Overall, the findings highlight that storyline negatively affects short-term game success, as customers, in general do not prefer radical changes with respect to storyline and setting of video games. This goes in line with the confirmation\disconfirmation paradigm as the customers expect video games with storylines similar to those already published on the market and thus is dissatisfied which leads to a negative impact on game success (Herrmann et al., 1999; Homburg & Stock-Homburg, 2006).

### Managerial implications

From a managerial perspective, several areas where innovation in game lead to an increase in video game sales could be identified.

First of all, as the results clearly depict, presentation is the most effective element in the short-term perspective, but does not play a role for long-term game success. Consequently, innovating presentational aspects of video games might offer good possibilities to enter new market segments or even new markets by temporarily satisfying the players’ needs for good graphics and sound (Hofacker et al., 2016; King et al., 2010; Wood et al., 2004). However, as Moore’s law predicted, the performance of computers or game consoles is continuously increasing (Moore, 1965). Every few years new generations of gaming platforms, computers and consoles alike, appear on the market with enhanced technical capabilities (Cadin et al., 2006; Gallagher & Park, 2002; Marchand & Hennig-Thurau, 2013). As the graphical and auditory elements of presentation are steadily increasing in their performance (Paterson et al., 2010; Situmeang et al., 2016), good presentation has only a positive impact on short-term success because in the long-term perspective it is already outdated and replaced by new technical possibilities after several months. For example, the development of deeper resolution lead from the introduction of HD-standard to UltraHD in only a few years (ITU, 2012), which is currently the best high-resolution standard in the entertainment industry. To conclude, managers of game producing companies should on the one hand focus on continuously creating new games based on new technical capabilities to not lose touch to the market level of presentational performance, while on the other hand they should keep in mind that innovating presentational aspects will not have an impact on the long-term success of the games and hence not on company performance either.

Second, the results also show that along with presentation, the degree of innovativeness of the game principle also positively influences short-term success. Hence, for producer and inventors of games it makes sense to increase their efforts of introducing good innovative game principles as they also have an impact on the short-term success of games. However, as with all innovative products, the danger of being copied, adapted or even further improved by other competing companies in the market exists (Koellinger, 2008). Hence, while producing games with innovative good game principles might often result in games that appear to be ageless, meaning that these games will always be played by some customers despite how old they might already be, this also implies them being copied and published under other titles by other companies. Examples for prominent ageless video games are ‘Tetris’ (1984; Engadget, 2019), ‘Pac-Man’ (1980; Suominen, 2012) and ‘Minesweeper’ (1989; Suominen, 2012) – three different games, produced between 1980 and 1989 but still being played under other titles (developed by other companies) on modern platforms like smartphones or other modern devices as of today (Suominen, 2012; Chan, 2008). Nevertheless, the focus on the degree of innovativeness of the game principle also offers the possibility to find and create new game types. An example offers the introduction of the Wii motion controller or the Playstation eye toy, which lead to the creation of motion games and opened up another possible market segment with new potential customers (Pasch et al., 2009). Overall, from a managerial perspective, focusing on the degree of innovativeness of the game principle makes sense, as long as it is not the only unique selling proposition, as this will not withstand the adoption of competitors for long.

Third, another important managerial implication of the study’s findings is about the degree of innovativeness of the storyline. Surprisingly, the study’s results reported a negative impact on short-term game success, which is equalized in the long-term perspective indicating that customers seem to be more attracted to games with a familiar storyline instead of a new innovative one. Additionally, the results might also indicate that games with a very innovative storyline have been produced more for a niche of specific consumers but not for the mass market of entertainment games hence only attracting a smaller number of interested players. Example for niche games are ‘Battles in Normandy’ (Strategic Studies Group) or ‘Achtung Panzer: Kharkov 1943’ (Paradox Interactive) – both games in a World War II setting. Taking all of this into consideration, game-producing companies should be careful during the development of new games with new storyline concepts. In line with other industries that already utilize the concept of customer co-creation and thus include customers today actively in the development of new products or services (Cova et al., 2011; Kleijnen et al., 2007), some video games are already being developed with support from active players rather than just by game developers alone (Jeppesen & Molin, 2003).

In summary, although it seems to be crucial to produce innovative games, managers should keep in mind that only a video game with the right level of innovativeness can result in market success in the long-term (Schultz et al., 2013).

### Limitations and directions for future research

When interpreting the results of this study, some limitations of the corresponding research design should be kept in mind. First, the study was conducted in the German gaming market, considering only games published in Germany between 2010 and 2015. As previous research has clearly shown, preferred game characteristics might differ worldwide as also other entertainment products, like motion pictures, are evaluated differently in various regions (Craig et al., 2005; Hennig-Thurau et al., 2007; Koban & Bowman, 2020). Subsequently, this leads to the opportunity to verify this research model in a cross-cultural setting to further strengthen external validity. Second, although many of the games included in our data set are also available on other platforms apart from PCs, the research was focused on the computer gaming market, as it still is the dominant platform for gamers in Germany (Digi-Capital, 2018). Nevertheless, console gaming is gaining ground and console games slightly differ from PC games, e.g. handling and control within the games (Tschang, 2005). Moreover, the study results showed that for computer games the innovative power of presentation has no significant impact on long-term game success as the technical capabilities for computers are evolving continuously. However, although game console producers are also continuously working on improving the performance of their devices, the general lifecycle of game consoles are around six years (Cadin et al., 2006; Gallagher & Park, 2002; Marchand & Hennig-Thurau, 2013) thus slightly longer than for computers. Therefore, research concerning game innovativeness in the area of video games could benefit from conducting this analysis in the area of console games.

Besides giving some ideas about addressing limitations of this study in future research, we subsequently outline some possible future research avenues. In recent years, the video game industry was characterized by a noticeable increase in mobile gaming, thus offering a great research potential (Marchand & Hennig-Thurau, 2013; Nam & Kim, 2020). Likewise, game-producing companies have noticed this change of platform and recognized the opportunity to enter a new market segment of video games. For example, in 2016 Nintendo produced a gaming app for smartphones based on the existing game series “Pokémon Go”. Within several weeks, the game became a great success and lead to a share value increase for Nintendo of more than 50% (The Independent, 2016). Consequently, a future research avenue would be to execute this analysis for the mobile gaming segment to check if different characteristics with degrees of innovativeness are relevant for the success of mobile games. Furthermore, it is possible that additional game characteristics typical for mobile games need to be considered, for example minimization of game elements to fit smaller displays or different control handling, to fully capture the degree of innovativeness for different gaming segments, hence rounding off the innovation research literature. Moreover, recent developments in video game motion controlling, such as the Xbox Kinect (Talaei-Khoei & Daniel, 2018), or augmented reality (Ellis et al., 2020; Laato et al., 2021) suggest that there might be an additional dimension of game innovativeness, focusing on the way video games are played. Future research might investigate whether and how the degree of innovativeness in gaming controls might affect video game success. Finally, during the COVID-19 pandemic millions of people were ordered to self-quarantine at home (Zhu, 2021). As a result, many people that previously had no contact with video games switched into casual gaming or even became regular gamers. This development especially counts for online gaming, where initiatives such as #PlayApartTogether promoted gaming for socializing and stress reduction during the COVID-19 pandemic (King et al., 2020). It would be interesting to investigate whether the relative importance of different types of game innovativeness may have changed during the COVID-19 pandemic. Future research might thus look into sales numbers and their connection to game innovativeness of video games published during the COVID-19 pandemic and compare the results to those found within our data set prior to COVID-19 pandemic. Apart from future investigations into performance effects of game innovativeness, it might be also interesting to take a look into how different types of game innovativeness evolve in new product development processes. As mentioned in the conceptual development section, product innovativeness “presumes a degree of creativity in the new product ideation and design processes” (Sethi et al., 2001, p. 74), but also other factors such as technology or functionality play an important role (Valgeirsdottir et al., 2015). Within this respect, it seems reasonable to assume that the relative importance of creativity and other factors to reach game innovativeness varies with respect to the type of game innovativeness. More specifically, presentation innovativeness seems most significantly linked to technological aspects such as visual progress, while creativity may not that often play the most important role in this regard. Yet, it still can be the main driver for presentation innovativeness as documented by games like Minecraft or Superhot VR, that stand out from the crowd due to their creative visuals. When it comes to storyline and game principle innovativeness, however, creativity might regularly play the most important role within new product development processes to reach these types of game innovativeness. Future research might shed light on this issue by investigating the relative importance of creativity in order to reach different types of game innovativeness.

## Conclusion

In various industries innovativeness has been identified as antecedent of competitive edges and financial performance in the long run (Gunday et al., 2011; Hubert et al., 2017; Ngo & O'Cass, 2013; Storz, 2008). Yet, empirical studies focusing on game innovativeness as success factor in the video game industry are still missing. As a consequence, this study strived to investigate whether and how the degree of innovativeness of specific game characteristics positively impacts short-term and long-term game success. The results from structural equation modeling demonstrate that the degree of game innovativeness of selected game characteristics, namely presentation, game principle and storyline, only influence short-term game success. While presentation and game principle positively influence short-term game success, storyline negatively impacts game success. The corresponding findings fill the current research gap and offer further insights concerning important innovational aspects of games to increase the success rate.

## Supplementary information

ESM 1 (DOCX 82 kb)

## Footnotes

1 The original procedures applied to our initial expert raters (3 instead of 8) was refined during the revision of this manuscript, such that the original structural model results somewhat deviated from those based on the new set of expert raters with the refined procedures reported in this manuscript. Corresponding results based on the initial expert rater team can be found in Online Appendix B.
