# Supplementary material for: Innovate or game over? Examining effects of product innovativeness on video game success
Source: Electron Mark. 2022 Mar 1;32(2):987–1002. doi: 10.1007/s12525-022-00521-7 (PMC8886200; doi:10.1007/s12525-022-00521-7)
Supplement: Supplementary file 1 — (DOCX 82 kb) [file 12525_2022_521_MOESM1_ESM.docx]

**Online Appendix**

**Appendix A:** Release dates of video games

| **Release Dates** | **2010** | **2011** | **2012** | **2013** | **2014** | **2015** | **Total** |
| --- | --- | --- | --- | --- | --- | --- | --- |
| 1st Quarter | 22 | 18 | 9 | 12 | 8 | 8 | 77 |
| 2nd Quarter | 16 | 16 | 9 | 8 | 16 | 10 | 75 |
| 3rd Quarter | 15 | 16 | 10 | 15 | 9 | 9 | 74 |
| 4th Quarter | 25 | 21 | 11 | 16 | 16 | 4 | 93 |
| **Total** | **78** | **71** | **39** | **51** | **49** | **31** | **319*** |

*For 32 video games it was not possible to find out the exact release date.

**Appendix B:** Procedures and Results based on the initial expert raters

All games were evaluated by three independent expert judges – common practice in marketing and innovation research - concerning their degree of innovativeness of presentation, game principle and storyline using the respective measurements. Each expert evaluated each game by means of the selected items using the following provided information: cover pictures, the description texts, the developer and brand of each game. The final value of the construct’s ‘game innovativeness’ was calculated by building the average of all expert opinions, making sure that no statistical outliers manipulated the results of this study. The corresponding structural model results based on the initial expert rater assessment can be found in Figure B1.


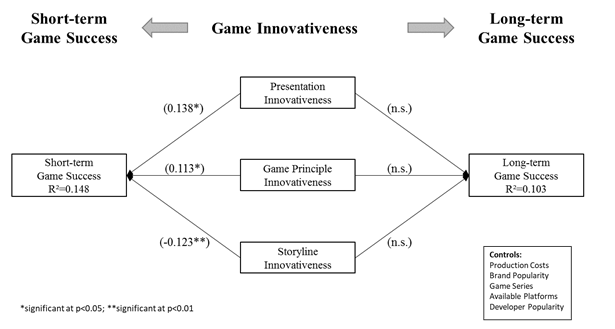


Figure B1: Structural model results based on initial expert rater assessment

As can be seen in Figure B1, we were able to replicate our findings with the new expert rater team and the revised procedures. All relationships that were found significant with our initial expert rater team were again confirmed. Moreover, the results turned out more robust and significant at least at 5% level. In addition, the previously positive but insignificant effect of game principle innovativeness on long-term success has also turned out significant at 5% level of significance. As our second expert team was much bigger than our first one (8 compared to 3 expert raters), the methodological procedures of the rating process were improved according to the reviewer comments, and the resulting model turned out more robust, we decided to include the corresponding results in our final manuscript instead of those with the initial rater team.

**Appendix C:** Additional Analysis

Contrary to our expectations, we found a negative effect of storyline innovativeness on short-term success which may have evolved due to gamers' expectations of incremental changes as discussed within our manuscript. However, we also thought about distinctive elements in video game types that might help explain our contradictory finding and further substantiate our arguments surrounding status quo tendencies. One of the principal elements distinguishing video game types is how players take decisions within games because games are built upon this aspect. Within this respect, affective and cognitive reactions are at the core of a person’s decision making (Shiv and Fedorikhin, 1999). While affective reactions are often just sensory-motor reactions, which occur relatively automatic, cognitive reactions require processing the given information and weighting the pros and cons of each alternative (Shiv and Fedorikhin, 1999). Consequently, one could assume that in games where affective reactions dominate (i.e. shooter), deviations from the status quo in terms of storyline innovativeness will be rather unnoticed due to low level of information processing, and thus not negatively affect the gaming experience and video game sales. However, in games where cognitive reactions dominate (i.e. strategy) such deviations will get noticed due to the high level of information processing and thus negatively affect the gaming experience and video game sales. Based on these arguments, we run a two-way ANOVA to compare the effect of storyline innovativeness for shooters to that of strategy games. Prior to evaluation, video games were classified into two groups according to their storyline innovativeness level by employing a median split at 3.58. Unfortunately, the interaction effect between level of storyline innovativeness (low/high) and game type (shooter/Strategy) on short-term game success deposited in Figure C1 turned out insignificant (F(3, 116) = 0.23, p = 0.879).


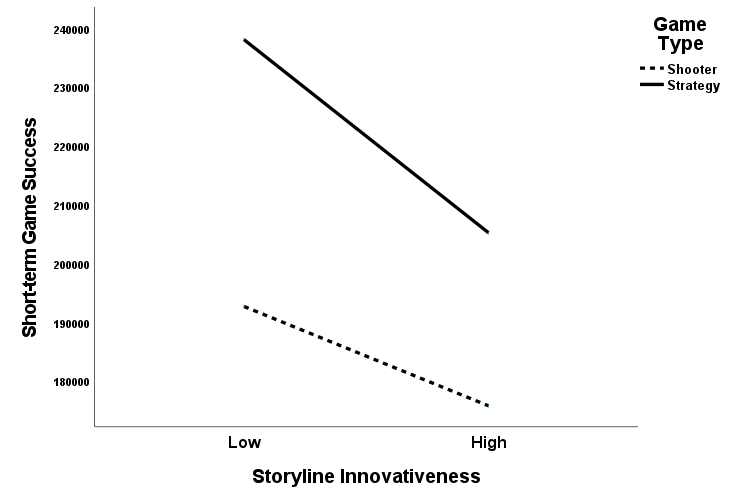


References

Shiv, B., Fedorikhin, A. (1999). Heart and mind in conflict: The interplay of affect and cognition in consumer decision making. Journal of consumer Research, 26(3), 278-292.
